# IHC Profiler: An Open Source Plugin for the Quantitative Evaluation and Automated Scoring of Immunohistochemistry Images of Human Tissue Samples

**DOI:** 10.1371/journal.pone.0096801

**Published:** 2014-05-06

**Authors:** Frency Varghese, Amirali B. Bukhari, Renu Malhotra, Abhijit De

**Affiliations:** Molecular Functional Imaging Laboratory, ACTREC, Tata Memorial Centre, Kharghar, Navi Mumbai, India; Health Canada and University of Ottawa, Canada

## Abstract

In anatomic pathology, immunohistochemistry (IHC) serves as a diagnostic and prognostic method for identification of disease markers in tissue samples that directly influences classification and grading the disease, influencing patient management. However, till today over most of the world, pathological analysis of tissue samples remained a time-consuming and subjective procedure, wherein the intensity of antibody staining is manually judged and thus scoring decision is directly influenced by visual bias. This instigated us to design a simple method of automated digital IHC image analysis algorithm for an unbiased, quantitative assessment of antibody staining intensity in tissue sections. As a first step, we adopted the spectral deconvolution method of DAB/hematoxylin color spectra by using optimized optical density vectors of the color deconvolution plugin for proper separation of the DAB color spectra. Then the DAB stained image is displayed in a new window wherein it undergoes pixel-by-pixel analysis, and displays the full profile along with its scoring decision. Based on the mathematical formula conceptualized, the algorithm is thoroughly tested by analyzing scores assigned to thousands (n = 1703) of DAB stained IHC images including sample images taken from human protein atlas web resource. The IHC Profiler plugin developed is compatible with the open resource digital image analysis software, ImageJ, which creates a pixel-by-pixel analysis profile of a digital IHC image and further assigns a score in a four tier system. A comparison study between manual pathological analysis and IHC Profiler resolved in a match of 88.6% (*P*<0.0001, CI = 95%). This new tool developed for clinical histopathological sample analysis can be adopted globally for scoring most protein targets where the marker protein expression is of cytoplasmic and/or nuclear type. We foresee that this method will minimize the problem of inter-observer variations across labs and further help in worldwide patient stratification potentially benefitting various multinational clinical trial initiatives.

## Introduction

Identification of various marker proteins in human tissue sample has been an important prerequisite in the clinical management of various diseases including cancer. Staining of various marker proteins located either in the cell nuclei, cytoplasm or membrane are often considered as pathological determinants for classifying and grading the disease. For identifying the presence and the extent of expression of such proteins, qualitative assessments is commonly done following techniques such as immunohistochemistry (IHC), immunocytochemistry (ICC) and immunofluorescence (IF) [1]. The basic underlying principle of these techniques is the staining of the biopsy tissue samples with antibodies specific to the molecular marker of interest. In IHC method, visualization of the antibody-antigen reaction is accomplished by the use of a secondary antibody conjugated to an enzyme, such as peroxidase, which catalyses a brown color-producing reaction. The processed sample slides are generally judged under a light microscope by a trained pathologist to assign a score based on the visual parameters set.

Originally, McCarty *et al.* (1986) developed the H scoring system [2], which was widely used until the introduction of a more recent, but a different scoring system by Allred *et al.* (1998) under the name of Allred or quick score [3]. Since both of these methods are manual, the issue of visual perception bias remains unanswered in addition to the time consumption which makes these methods low throughput to meet the growing need of large cancer hospitals. The existing clinical scoring process is based on two characteristics: overall stain intensity and the proportion of neoplastic tissue stained. The pattern of the stain is broadly categorized on the basis of the percentage of cells stained i.e. >75% – uniform; 25–75% – variable; and 0–25% – rare. The overall score of the staining intensity typically has four tiers ranging from 0 to 3 [4]. One major problem in determining the standard by this approach is the amount of variability due to visual perception on a hematoxylin counter-stained tissue section [5], [6].

With the introduction of advanced digital image processing systems, the emergence of a number of both, commercial as well as freely available computer-assisted softwares have been introduced in order to rally the high volume IHC analysis and scoring [1], [5], [7]–[14]. A majority of the modern cellular imaging systems are accompanied by proprietary software that offers a diversity of quantitative information about the acquired images, but in order to perform scoring calculations, users need to specify the intensity threshold and frequency of stained cell within the image areas. The choice and determination of threshold being a critical step for all subsequent quantification can itself be subjective and thus leads to a user-dependent discrepancy in tissue sample scoring. Additionally, the high cost of the commercially available softwares often limits the application of such automated IHC scoring in research organizations or hospitals. On the other hand, the available free tools are yet to arrive to a consensus depicting the accuracy standards. Only a few studies have compared the visual human interpretation to that of the computer aided vision of IHC expression levels with reverence to the clinically significant factors and endpoints, such as determining the outcome of a disease [14]–[17].

Keeping in view the above mentioned limitations of various analytical methods, we report here, the development of an open source plugin named IHC Profiler, which is compatible with the ImageJ software and demonstrate the method for IHC analysis using color deconvolution and computerized pixel profiling leading to the assignment of an automated score to the respective image. This comprehensive method demonstrated here has been thoroughly validated using high volume IHC digital dataset representing multiple protein markers which have shown either cytoplasmic or nuclear expression.

## Materials and Methods

### Ethics Statement

The clinical study protocol was reviewed and approved by the TMC-ACTREC Institutional Review Board. For several experiments paraffin embedded tissue blocks were obtained for use from our tumor tissue repository and thus patient consent waiver was obtained.

### The Human Protein Atlas

Immunohistochemistry images of various human tissue samples stained with a variety of marker protein (antibody) were also obtained from the human protein atlas (http://www.proteinatlas.org/) with permission [18], [19]. Scoring was performed and the data was matched for similarity.

### Immunohistochemistry

Standard IHC protocol was followed to stain the tumor tissue samples using the mouse monoclonal antibody against hNIS (human Sodium Iodide Symporter) (Abcam, ab17795), ER (Estrogen Receptor) (Abcam, ab16660, ab288). Briefly, 5 µm sized paraffin embedded tissue sections were de-paraffinized with xylene and endogenous peroxidase activity was quenched with 3% H_2_O_2_ in methanol for 30 minutes in the dark. Tissue sections were dehydrated through graded alcohols and subjected to antigen retrieval using 10mM sodium citrate. Sections were washed with TBST (Tris Borate Saline Tween-20) and then blocked with 5% BSA (Bovine Serum Albumin) for one hour. Slides were incubated with the respective mouse monoclonal primary antibody diluted with TBS. Slides were then washed for 5 minutes in TBST and incubated for 1 hour with the respective HRP (Horse Raddish Peroxidase) conjugated anti-mouse secondary antibody diluted with TBS in a ratio of 1∶200. After washing, slides were incubated with DAB (3,3′-diaminobenzidine tetrahydrochloride) (Sigma) and immediately washed under tap water after color development. Slides were then counter stained with hematoxylin. Slides were mounted with DPX (dibutyl phthalate xylene) and were then observed under a light microscope (Carl Zeiss).

### Image Acquisition

Images were captured using the Zeiss Imager.Z1 upright microscope (Zeiss, Germany) equipped with an AxioCam MRc5 camera (Zeiss, Germany), interfaced with an IBM Think Centre computer (International Business Machines Corporation, USA). Light and camera settings were controlled using the AxioVision V4.6 (Zeiss, Germany) software, resulting in average background values of 63±13 milliseconds (mean ± standard deviation) for the red, green and blue channels. Images were captured at 10X, 20X, and 40X objective lenses.

### Optical Density Vector Determination

To determine the correct optical density (OD) vectors for the RGB channel of Hematoxylin and DAB, we followed the protocol as previously described by Ruifrok *et al.* [1]. Since the optical density is proportional to the concentration of the stain, the amount of stain present will be a factor determining the optical density at a wavelength specific to the stain as per the Lambert-Beer law [20]. In brief, the OD for each channel is defined as,
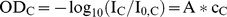
Wherein, OD is the optical density, I is the transmitted light and I_C_ being the intensity of the detected light after passing through the specimen, I_0,C_ is the intensity of light entering the specimen, and A is the amount of stain with an absorption factor c. The subscript c indicates the detection channel. The detected intensities of light transmitted through a specimen is as described by the Lambert-Beer's law [20].

### Image Analysis

The IHC images used were stained with DAB and hematoxylin. The result of color deconvolution leads to the production of three images, namely, DAB, hematoxylin and a complimentary image. In a previous study, we reported development of an ImageJ compatible plugin for analyzing cytoplasmic staining pattern by assigning a histogram profile for the deconvoluted DAB image [21]. Now, within the scope of the current plugin development, we envisioned automating the whole process by integrating deconvolution, histogram profiling and scoring by a simple choice of the program menu. Additionally, it integrates methods with a wider scope of analyzing various marker proteins displaying cytoplasmic or nuclear staining patterns (**Table 1**).

**Table 1:** List of the cancer samples and immunogens tested during the current study.

Cancer Type	Markers Analysed
*Breast cancer*	hNIS, ER, PR, p53, STAT3, Ki-67, BRCA1, BRCA2, VEGF, Cyclin D1
*Colon Cancer*	Lamin A/C, myc, VEGF
*Cervical Cancer*	STAT3
*Liver Cancer*	BPDE-DNA adducts, VEGF, Cyclin D1
*Lung Cancer*	Akt-ser, Akt-Thr, Bax, Bcl-2, BPDE-DNA adducts
*Melanoma*	BRAF, Fascin, MMP3
*Thyroid Cancer*	hNIS
*Oral Cancer*	Bax, Bcl-2, Cox2, PCNA, Survivin, Jnk, p38, p-Jnk, Akt, Vimentin, CK5, CK8, CK18
*Ovarian Cancer*	p53, HOXA9, HOXA10, HOXA13

In digital image analysis, the pixel intensity values for any color range from 0 to 255, wherein, 0 represents the darkest shade of the color and 255 represent the lightest shade of the color as standard. A total of 1703 images were analyzed independently with the help of two expert pathologists and were assigned a score as high positive (3+), positive (2+), low positive (1+) and negative (0). In the current method development, the next step was assigning a histogram profile which is a plot between the intensity values of the pixels (X axis) vs. the number of pixels representing the intensity (Y axis). Keeping in view the standard grading procedure, the histogram profile was divided into 4 zones, viz. high positive, positive, low positive and negative. These four zones were equally divided on the pixel color intensity bar (as indicated in **Figure S1A**).

The zones were visually identified by using the threshold feature of Image menu of the ImageJ program [22]. To begin with, the intensity values were grouped into bands of 10 and the corresponding regions in the image were confirmed by using the threshold feature. Thus initially all the intensities from 0 to 10 were turned red on an image with a known pathological score of high positive. Then, in addition to the previous band, the next band, from 11 to 20 were turned red and the same was continued until all the pixels of the brown shades were assigned a threshold and the range for the high positive zone was determined. Similarly, zone containing the lightest color shade of pixel intensities was also determined using an image with a known pathological score of negative. This was because once the highest intensity (high positive) and the least intensity (negative) zones were determined, it would help towards the better determination of the size of the intermediate (positive and low positive) zones. It was found that the region between 0 and 60 contained pixels of the high positive stained images. Similar was noted on samples with known pathological lower scores to optimize the correct range. The process was repeated for at least 70 images of same intensity of the color shade. The intensity range for the positive zone was found to be ranging from 61 to 120; 121 to 180 for the low positive zone; and 181 to 235 for the negative zone, respectively (**Figure S1**). It was determined that the pixels with intensity values ranging from 235–255 predominantly represent fatty tissues which are occasionally present but do not typically contribute to pathological scoring and were therefore excluded from the score determination zones. The intensity ranges determined visually were confirmed by plotting a histogram using Microsoft Excel (data not shown).

### Score Calculation

A simple algebraic formula was conceptualized for score assignment to the IHC images.
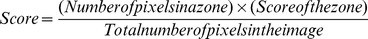
Wherein, the score of the zone is assigned as 4 for the high positive zone, 3 for the positive zone, 2 for the low positive zone and 1 for the negative zone. Also, any image containing 66% or more percentage of pixels in a zone are directly assigned a score of that zone, eliminating the need to apply the formula. However, in case an image lacks the majority (lesser than 66%) for any particular zone, the above formula will operate to determine the score.

### IHC Profiler

The current plugin named as IHC profiler, integrates options for quantitative analysis of digital IHC images stained for either cytoplasmic or nuclear proteins. Demonstration video to perform quantitative scoring analysis of the cytoplasmic stained sample (**Movie S1**) and that of the nuclear stained sample (**Movie S2**) can be found in supplementary data. IHC profiler can be freely downloaded from Sourceforge website (https://sourceforge.net/projects/ihcprofiler/). IHC profiler is currently compatible for use with Microsoft Windows operating system. Guidelines pertaining to the use of IHC profiler and embedding it to the Windows based ImageJ program can also be found in the package.

### Statistical Analysis

Statistical analysis was performed using GraphPad Prism 6 software (GraphPad Software, La Jolla California USA). To evaluate the agreement between the manual and automated scoring methods, we split the scores into the groups of high positive, positive, low positive, and negative. The significance of difference was obtained by performing the two tailed chi-square test and CI set at 95%. Value of *P* lesser than 0.05 were considered significant and that lesser than 0.001 were considered highly significant. For the comparison of the two grading methods, kappa statistical analysis was performed.

## Results

### Optical density vector optimization

**Figure 1:**
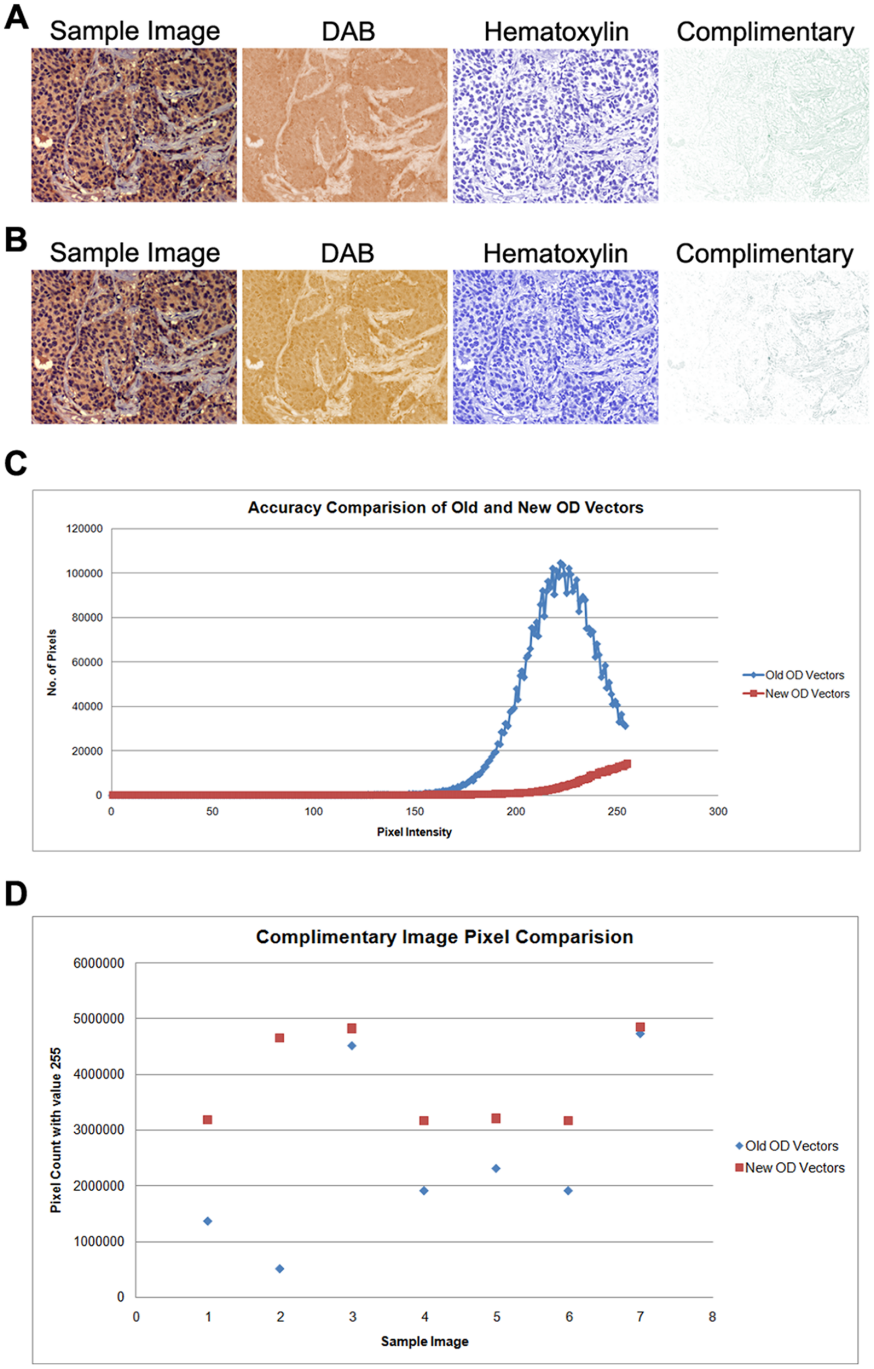
Representation of color deconvolution using the old and the new optical density (OD) vectors. **A:** Color deconvolution using the old OD vectors. **B:** Color deconvolution using the new OD vectors. **C:** Scatter plot comparing the intensities on the complimentary image with the old OD vectors (blue) and the new OD vectors (red). **D:** Plot comparing the number of pixels with the intensity value of 255. An improvement between 2 to 10 fold is shown using 7 different samples. Each data plot represents an individual sample with its respective pixel count of the intensity value of 255.

As a first step, we attempted color deconvolution of IHC images using the set optical density vectors for DAB and hematoxylin (H DAB as mentioned in the plugin) in the color deconvolution plugin for ImageJ developed by Ruifrok *et al.* [1]. When the two stains (i.e. blue color representing hematoxylin and brown representing DAB) were separated from an image, the third complimentary image, contained shades of both DAB and hematoxylin (**Figure 1A**). Therefore, in order to rectify we analyzed multiple different images of varying staining pattern to determine the correct optical density (OD) vectors for hematoxylin and DAB. The images produced upon color deconvolution by the new OD vectors are represented in the figure (**Figure 1B**). We observed a substantial improvement in intensity distribution of pixels on the complimentary images as represented in Fig. 1A and Fig. 1B (**Figure 1C**). This improvement was based on the correction of the vectors that resulted in the loss of a large amount of pixels in the complimentary image. To verify the utility and accuracy of the new OD vector in deconvoluting various antibody stained IHC samples, 147 different samples of cancer tissue stained with various antibodies were attempted. Intensity values were analyzed and compared for both, pre- and post-correction OD values. Finally, depending on the IHC staining procedure followed, a 2–10 fold variation in complimentary image was observed (**Figure 1D**).

### Automated image scoring for cytoplasmic and nuclear protein targets

**Figure 2:**
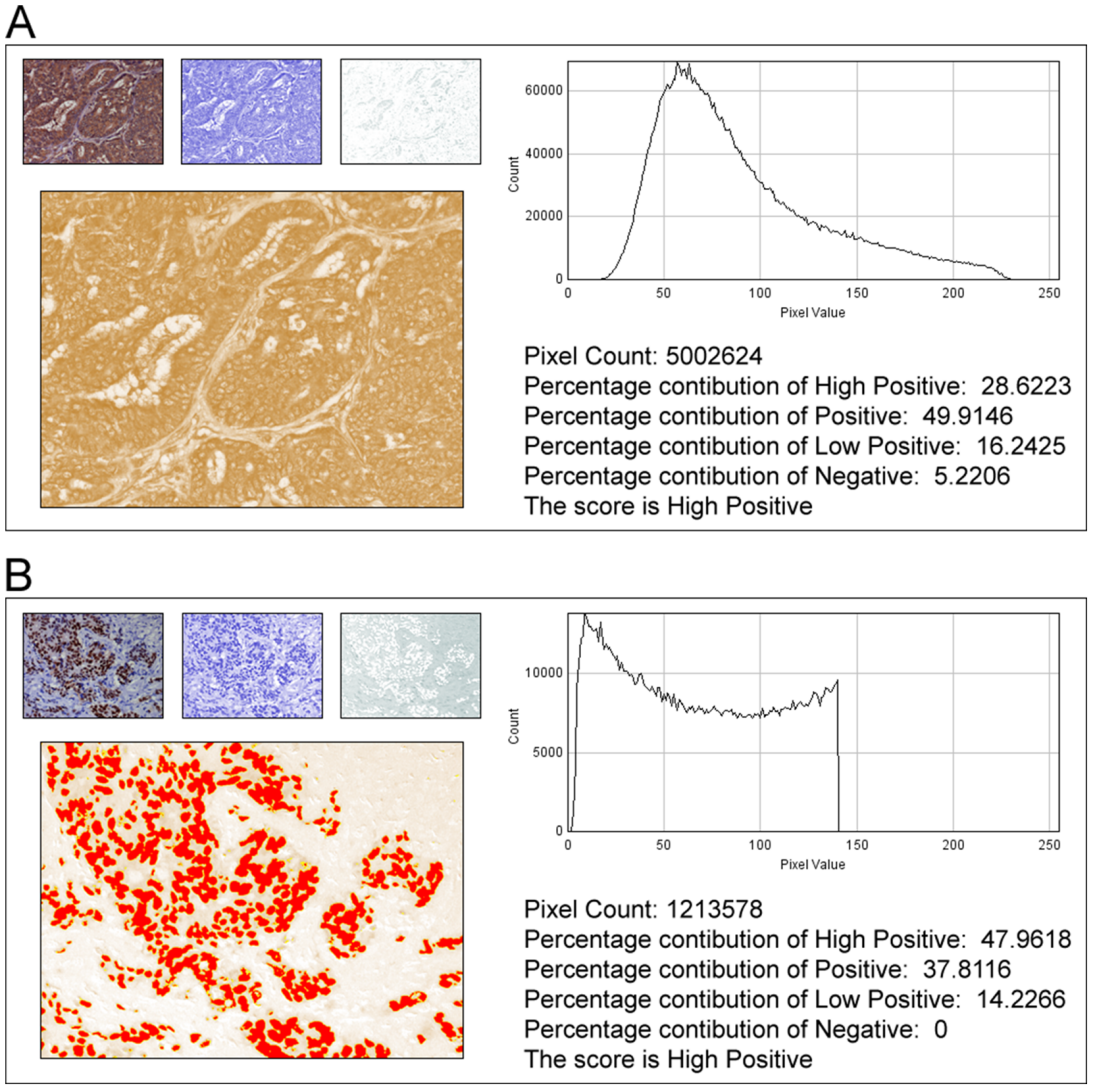
Representative histogram profile and score of a cytoplasmic and nuclear stained image using IHC Profiler. **A:** Profiling of the DAB stained cytoplasmic image sample. The histogram profile corresponds to the pixel intensity value vs. corresponding number counts of a pixel intensity. The log given below the histogram profile shows the accurate percentage of the pixels present in each zone of pixel intensity and the respective computed score. **B:** Profiling of the DAB stained nuclear stained image sample. The red spots on the DAB image indicate the threshold selection of the nucleus areas using the threshold function of ImageJ. The representative histogram profile corresponds to the number of pixels vs. the corresponding value at which the pixel of the respective intensity is present.

To automate the scoring process, we developed a new macro compatible for use with ImageJ program that generates a histogram profile of the DAB image and applies the scoring formula as described previously. This program named ‘IHC Profiler’ has been embedded under the plugin menu of the ImageJ software. The scoring method is optimized by analyzing over 800 stained IHC images with a predominantly cytoplasmic staining pattern. As a general procedure, images are opened in ImageJ, followed by deconvolution using the newly optimized color deconvolution plugin. With the selection of the ‘H DAB’ vector on the color deconvolution popup window, IHC profiler automatically plots a histogram profile of the DAB image and the corresponding scoring log is displayed on the screen as shown in **Figure 2A**.

**Figure 3:**
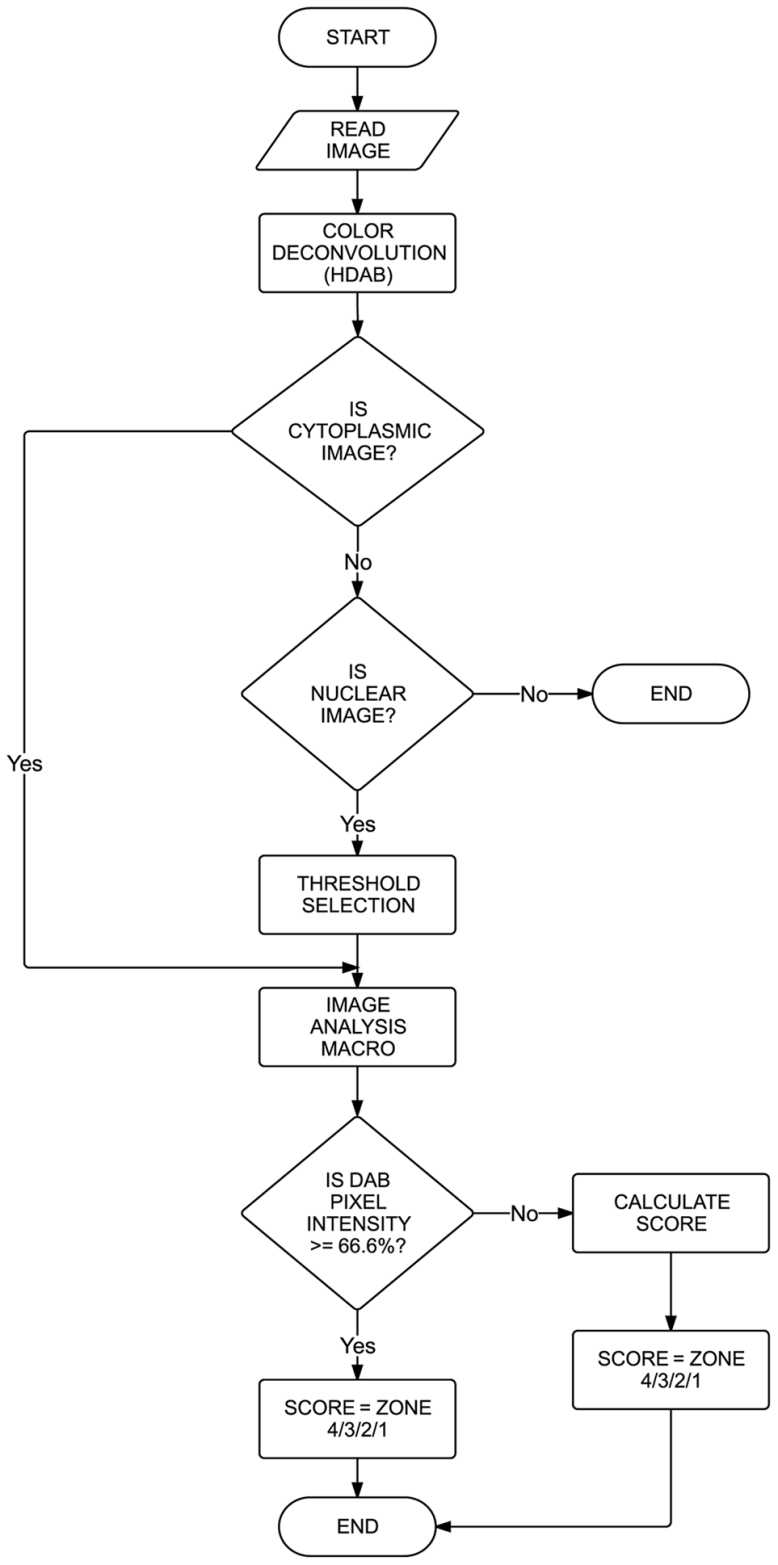
Flow chart demonstrating the computing steps involved in the working algorithm.

Similarly, for images with nuclear staining pattern, over 900 images are analyzed. As the deconvoluted DAB staining pattern for nuclear proteins are confined primarily to the nuclei, the ‘threshold’ feature of the ImageJ program is used to select the positively stained areas with brown stained pixels and further the ‘create selection’ option from image menu is used to mark a selection around them. Once this selection is applied on the image, IHC profiler assigns a histogram profile as well as its corresponding log to the respective image (**Figure 2B**). The flow chart representing the functioning of the algorithm designed for the macro is shown in **Figure 3**.

### Automated IHC scoring macro improves accuracy and decreases observer bias

**Figure 4:**
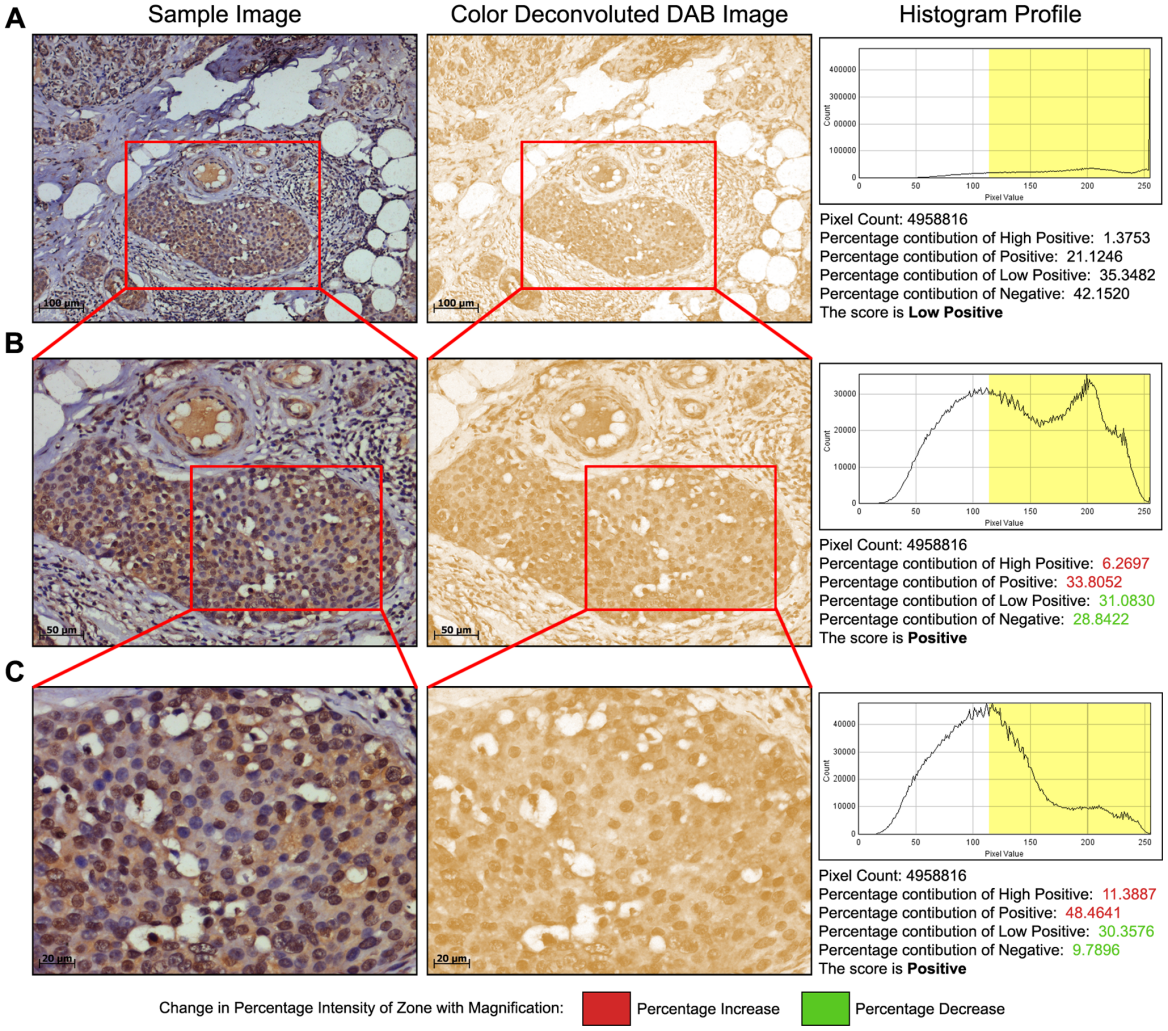
Impact of magnification on image scoring. **A:** Analysis of a 10X image area where a significant amount of stroma and fatty tissue is present. After color deconvolution, the score assigned by IHC profiler on the DAB image was determined as low positive. **B:** Scoring analysis of the same tissue area where image captured was by using a 20X lens in the marked area, focusing more on the actual tumor mass resolute a score of positive. **C:** Scoring analysis of the same tissue area wherein the image was captured using a 40X lens, focusing more on eliminating the stromal and fatty tissue region increases the percentage of the positive pixels in the positive and high positive zones.

By analyzing over 1700 cytoplasmic and nuclear stained IHC images, scores obtained using the IHC profiler are then compared with the scores assigned by expert pathologists. Our findings with the automated scoring macro resulted in an 88.6% match with that of the pathologically scored data (*P*<0.0001, CI  = 95%) (**Table 2**). Additionally, the agreement of scores between IHC profiler and the manual analysis process has been shown in **Table 3** (Kappa  = 0.843). Discrepancies in the verdict of a pathologist may vary that from one pathologist to another depending on his/her experience in the discipline. To achieve stronger confidence in our findings, we added a third pathologist to the study. The strength of agreement in the inter-observer comparisons reveal that the opinions of observer 1 and 2 is found to be worse than what one would expect to see by chance alone (Kappa  = −0.669) (**Table 4**) and that in between observer 2 and 3 is considered to be ‘poor’ (Kappa  = 0.011) (**Table 5**). However, the strength of agreement is considered to be ‘good’ in between observer 1 and 3 (Kappa  = 0.715) (**Table 6**). Further accuracy analysis shows that majority of the cases where the automated IHC scoring differs by 1 or 2 degrees from that of the manual scoring, are mostly the cases where the ratio of tumor tissue to stromal tissue is low. This is mainly because a high percentage of pixels present in stroma represents low intensity values (pixel value <200), and are responsible for a lower average score thus assigned. However, when a pathologist reads the slide, they ignore stromal staining and assigns a score value primarily based on tumor tissue staining intensity. In an automated process, this is difficult to achieve and may require manual supervision. To address this issue in 15–20% cases where low tumor to stroma ratio is observed, we applied zoom, wherein images captured using higher magnification are used thereby showing significant improvements in achieving correct pathological scoring (*P* = 0.002, CI  = 95%) (**Figure 4**).

**Table 2:** Comparison chart showing automated vs. manual scoring.

**Total number of high positive (3+) cases**	312
**Total number of positive (2+) cases**	481
**Total number of low positive (1+) cases**	572
**Total number of negative (0+) cases**	338
** Total number of cases studied**	1703
**Number of cases where inter-observer score does not match**	383
**Number of cases where inter-observer scores match but differ from automated analysis**	150
**Number of cases where automated scores and manual scores differ due to higher stroma to tumor ratio**	124
**Percentage match between manual and automated scoring before stroma to tumor ratio corrections by higher magnification**	77.5%
**Percentage match between manual and automated scoring after stroma to tumor ratio corrections by higher magnification**	88.6%

Table shows the distribution of samples and a comparison study between the automated and the manual scoring. Total number of cases determines the sample size taking into account for the study. The difference of significance was obtained by two-tailed chi-square test resulting into values of *P*<0.0001 (CI  = 95%).

**Table 3:** Agreement of scores between manual vs. IHC Profiler assessment.

	Manual					
		High Positive	Positive	Low Positive	Negative	Total
**IHC Profiler**	**High Positive**	250	22	0	0	**272**
	**Positive**	17	416	43	0	**476**
	**Low Positive**	0	39	327	16	**382**
	**Negative**	0	1	12	177	**190**
	**Total**	**267**	**478**	**382**	**193**	**1320**

Table summarize the agreement of scores between the manual scoring process vs. IHC profiler assessment. This table excludes the samples wherein the inter-observer score did not match with each other. Kappa statistics was performed and the value of Kappa  = 0.843 (95% CI: From 0.819 to 0.867).

**Table 4:** Variability of scores in between the pathological opinions.

	Observer 2					
		High Positive	Positive	Low Positive	Negative	Total
**Observer 1**	**High Positive**	0	0	0	0	**0**
	**Positive**	0	0	52	0	**52**
	**Low Positive**	0	48	0	153	**201**
	**Negative**	0	0	127	0	**127**
	**Total**	**0**	**48**	**179**	**153**	**380**

Table summarizes the inter-observer variability of two pathologists whose opinions were taken into consideration during this study (383 cases as shown in Table 2). Sample number was rounded off to 380 for statistical comparison between the two groups. Kappa statistics was performed and the value of Kappa  = −0.669 (95% CI: From −0.702 to −0.637) indicates the strength of agreement is worse than what one would expect to see by chance alone.

**Table 5:** Variability of scores in between the pathological opinions.

	Observer 3					
		High Positive	Positive	Low Positive	Negative	Total
**Observer 2**	**High Positive**	0	0	0	0	**0**
	**Positive**	0	0	07	0	**07**
	**Low Positive**	0	12	111	57	**180**
	**Negative**	0	0	123	70	**193**
	**Total**	**0**	**12**	**241**	**127**	**380**

Table summarizes the inter-observer variability of two pathologists whose opinions were taken into consideration during this study (383 cases as shown in Table 2). Sample number was rounded off to 380 for statistical comparison between the two groups. Kappa statistics was performed and the value of Kappa  = 0.011 (95% CI: From −0.078 to 0.099) indicating the strength of agreement is considered to be ‘poor’.

**Table 6:** Variability of scores in between the pathological opinions.

	Observer 3					
		High Positive	Positive	Low Positive	Negative	Total
**Observer 1**	**High Positive**	0	0	0	0	**0**
	**Positive**	0	33	02	0	**35**
	**Low Positive**	0	17	173	37	**227**
	**Negative**	0	0	07	111	**118**
	**Total**	**0**	**50**	**182**	**148**	**380**

Table summarizes the inter-observer variability of two pathologists whose opinions were taken into consideration during this study (383 cases as shown in Table 2). Sample number was rounded off to 380 for statistical comparison between the two groups. Kappa statistics was performed and the value of Kappa  = 0.715 (95% CI: From 0.651 to 0.778) indicating the strength of agreement is considered ‘good’.

## Discussion

Arriving at a common consensus in the quantitative analysis of immunohistochemistry images is still an issue in clinical pathology. Since the IHC slides are predominantly stained with DAB and are counterstained with hematoxylin, discrepancies in the verdict of the accurate color intensity turns out to be a problem and thus often leads to inter-observer variations. Assessment of the percentage of areas stained has a varying (poor to good) rate of reproducibility [23], even if the derived data by a pathologist have good to excellent inter-observer reproducibility [23], [24].

Due to the above mentioned limitations of visual estimation, means of automated quantitation of IHC images may provide the necessary detailing to improve IHC data quality across the globe. This is important as the practice of clinical decision making processes across the world will ensure best patient benefit. Numerous models have been presented using diverse systems of image color modes. Apart from quantitative image analysis by color deconvolution [1], there have been reports pertaining the use of the red-green-blue (RGB) method [9], the cyan-magenta-yellow-black (CMYK) method [13], the hue-saturation-intensity (HSI) method [25], and the CIE Luminance U-chromatic component 1 V-chromatic component 2 (CIELUV) method [17]. Despite the fact that these studies provided with reasonably good results in chromogen discrimination over the background, most of them, however, make use of rather sophisticated softwares, and lengthy protocols and algorithms which make them complicated for routine use in clinic and research laboratories handling large volume of patient samples.

In this study, we therefore focused on developing an automated portal linked to a open source analysis software (i.e. ImageJ) to quantify the staining on IHC images which requires minimal supervision for analysis of both cytoplasmic and nuclear protein markers in patient sample cases. The proposed algorithm results in the assignment of a score along with a detailed histogram profile of the image on the basis of the pure DAB stain intensity obtained through color deconvolution. The currently available color deconvolution procedure by Ruifrok *et al.* [1] majorly takes the stain separation into account and does not emphasize more on the pixels lost in the complimentary image generated. This generally results in a false positive stain separation of DAB and thus may lead to improper judgment of the score value. The main advantages of the method developed are that, **i.** it is compatible with an open source and widely used digital image analysis program ImageJ unlike a few tools that require a separate platform to perform the desired operation [5], [11]; **ii.** requires only the sample image photographs and returns a full pixel analysis report of the entire image area; **iii.** eliminates inter-observer visual perception bias; **iv.** can significantly improve the throughput by reducing the time burden of high volume sample analysis compared to that of traditional manual scoring process and distinct from the quantitative image analysis study by Tuominen and colleagues wherein the higher volume of samples can be a hurdle in the time consumed [11]; **v.** simple, requiring only couple of steps to follow to analyze each sample through it; **vi.** significantly eliminates the severe dependency on a trained pathologist contrasting reports by Turbin *et al.* and by Gokhale *et al.* wherein inputs from a trained pathologist are crucial [26], [27]. Alternatively, the vast applicability of IHC profiler helps it stand in line with the “gold standard” commercially available tools (**Table 7**). IHC profiler is independent of the need to select a threshold where the marker protein is cytoplasmic in nature. However, in cases where the marker protein is nuclear by nature, scoring the entire image will obviously lead to inaccurate results, a secondary step has been added to make use of ImageJ program's threshold feature in a user defined manner to select the DAB stained nuclear areas and mark them by the ‘create selection’ tool to specify areas for automated analysis. By testing several hundreds of samples of each type, we obtained good confidence on the assigned score.

**Table 7:** Comparison of IHC profiler with available IHC image analysis tools.

Software Name	Pros	Cons
**IHC Profiler**	• Freely available• Open source, ImageJ compatible• Easy to learn and use• Bias-free analysis• Time saving (takes about 1–2 min to analyse an image depending on the user experience)• Compatible with various image file formats (JPEG, PNG, TIFF, BMP)• Can analyse both cytoplasmic and nuclear stain immunomarkers• Can be used for the analysis of wide-range of markers and cancers• Users capable of analysing the whole image or a region of interest	• Analysis do not support membrane immunomarkers with stains in the cell membrane• May require an experts supervision for identification of non-neoplastic cells and tissue necrosis
**ImmunoRatio**	• Freely available• Web based application and runs within the web browsers• Bias-free analysis• Users capable of analysing the whole image or a region of interest• Compatible with various image file formats (JPEG, PNG, TIFF, BMP)• Cross-platform compatibility• Available with two modes of analysis (basic and advanced)	• Time consuming for larger sized image files• Can analyse only nuclear immunogen staining• Demonstrated capability of quantitation of ER, PR, and Ki-67 markers for breast cancer
**TissuemorphDP**	• Can be used for the analysis of multiple markers and cancers• Can analyse membrane, cytoplasmic, and nuclear stain immunomarkers• Bias-free analysis• Uses apps for analysis of various immunostains• Can analyse the entire slide at the same time	• Commercial, cost-effective• Additional cost for purchasing apps• Time consuming (analysis time of 10–20 min/image depending on user experience)• May require an experts opinion for identification of non-neoplastic cells and tissue necrosis• Requires whole slide scanner• Requires dedicated learning/learning time and customer support

Further, an accuracy comparison study was also performed, which designate that 88.6% of the scoring assigned by IHC profiler are in fair agreement with blinded manual scoring by pathologists (*P*<0.0001, CI  = 95%) (**Table 2**). Keeping in view the biological variations in human tissue samples, unsupervised scoring analysis demonstrating such a high percentage match with the traditional analysis would seem to be an important achievement. Additionally, a lot of previously demonstrated softwares were confined to score only specific target proteins and were not verified accuracy for a wide spectrum of biological targets and/or samples [11], [14], [16], [17], [28], [29]. We have demonstrated the use of our algorithm on various cancer types and several protein targets with their expressions confined either to the cytoplasmic region or the nucleus region (**Table 1**). The demonstrated scoring capacity of the current method would thus help to achieve a universal standard in IHC scoring (**Figure S2**). Furthermore, of all the cases where the scores differ by 1 or 2 degrees are generally those where tumor to stroma ratio is low. In cancer tissue samples, this type of variation is quite common, where experts reading the slide ignore the stromal staining and consider only the staining intensity of the tumor cells. For such cases, a manual judgment decision to use higher magnification images (40X) for analysis will certainly provide a more accurate score as this minimizes the averaging effect. Alternatively, an averaging method can also be attempted, where multiple images captured at different field of view of the same sample can help to achieve a true reflection for the entire tissue section. Additionally, studies concerned with the grading of the nuclear region also recommend an image taken using a 40X objective due to the small size of the nucleus and the need to select the threshold manually. Furthermore, it is important to note that the pixel count of each image scored can vary depending on the resolution of the camera used to capture the image, and this variation does not bias the scoring decision as it is not a bar to obtain a uniform score (**Figure S2**).

Staining of non-neoplastic cells, problems concerning with tissue necrosis, uneven fixation of the tissue samples, etc., are amongst the technical errors which is beyond the scope of the IHC profiler method. Such cases would also require the opinion of a trained pathologist in order to identify the neoplastic region. Future developments in automated digital image capture, making it compatible to the Macintosh (Apple Inc.) and Linux operating systems, and modifications in the method to adapt it for the receptor proteins being expressed on the cell boundaries only are possible. Forthcoming analysis systems may also facilitate an automated evaluation of the whole-tissue sections by integrating this method with software controlled stage movement and thereby dramatically reducing the time involvement in accumulating and evaluating images.

## Conclusion

This study demonstrates development and utility of an open source plugin that is ImageJ compatible towards automating quantitative IHC analysis and scoring. IHC Profiler can be utilized in the screening of various prognostic biomarkers across various types of cancers and normal tissues. The analysis method developed demonstrated high confidence with manual pathological analysis, and thus we conclude that this method holds the potential in developing fast and unsupervised analysis of IHC slides in various clinical and research laboratories.

## Supporting Information

Figure S1 **Different zones assigned for the scoring of the DAB stained image.** **A:** Shows the reference bar distributing the various zones ranging from 0 to 235. 235 to 255 pixel values are generally found to represent fatty tissues or blank areas and thus kept out of range for zone considerations. **B:** High positive (3+) image with its corresponding reference bar. **C:** Positive (2+) stained image with its corresponding reference bar. **D:** Low positive (1+) stained image with its corresponding reference bar. **E:** Negative (0) stained image with its corresponding reference bar.
(TIF)

Figure S2 **Demonstration of wide applicability of IHC profiler in various cancer and normal tissue types.** Respective image analysis output and the score assigned using IHC Profiler is also shown for each image. Duly note, the varying number of pixels is solely due to the resolution of the microscope camera at which they were captured.
(TIF)

Figure S3 **Qualitative measurement of IHC profiler accuracy.** Qualitative measurement of IHC profiler accuracy and its comparison with pathological analysis post assessment of IHC profiler. Red arrow markings indicate overdeveloped regions of the sample as indicated by pathological assessment.
(TIF)

Movie S1 **Scoring of a sample cytoplasmic stained image using IHC profiler.**
(AVI)

Movie S2 **Scoring of a sample nucleus stained image using IHC profiler.**
(AVI)
