# Supplementary figures and images for: IHC Profiler: An Open Source Plugin for the Quantitative Evaluation and Automated Scoring of Immunohistochemistry Images of Human Tissue Samples
Source: PLoS One. 2014 May 6;9(5):e96801. doi: 10.1371/journal.pone.0096801 (PMC4011881; doi:10.1371/journal.pone.0096801)

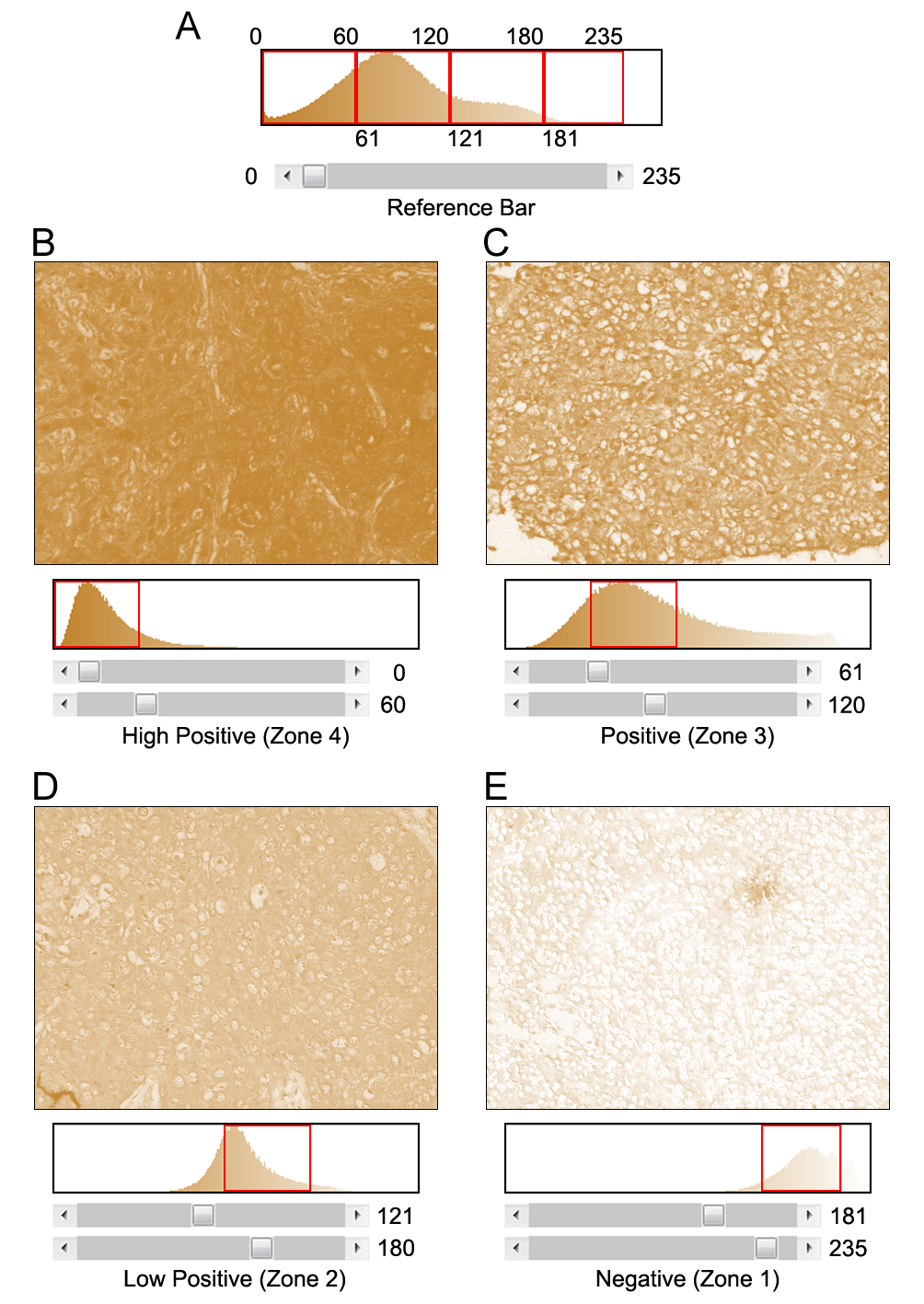

Supplement: Figure S1 — Different zones assigned for the scoring of the DAB stained image. A: Shows the reference bar distributing the various zones ranging from 0 to 235. 235 to 255 pixel values are generally found to represent fatty tissues or blank areas and thus kept out of range for zone considerations. B: High positive (3+) image with its corresponding reference bar. C: Positive (2+) stained image with its corresponding reference bar. D: Low positive (1+) stained image with its corresponding reference bar. E: Negative (0) stained image with its corresponding reference bar. (TIF) [file pone.0096801.s001.tif]

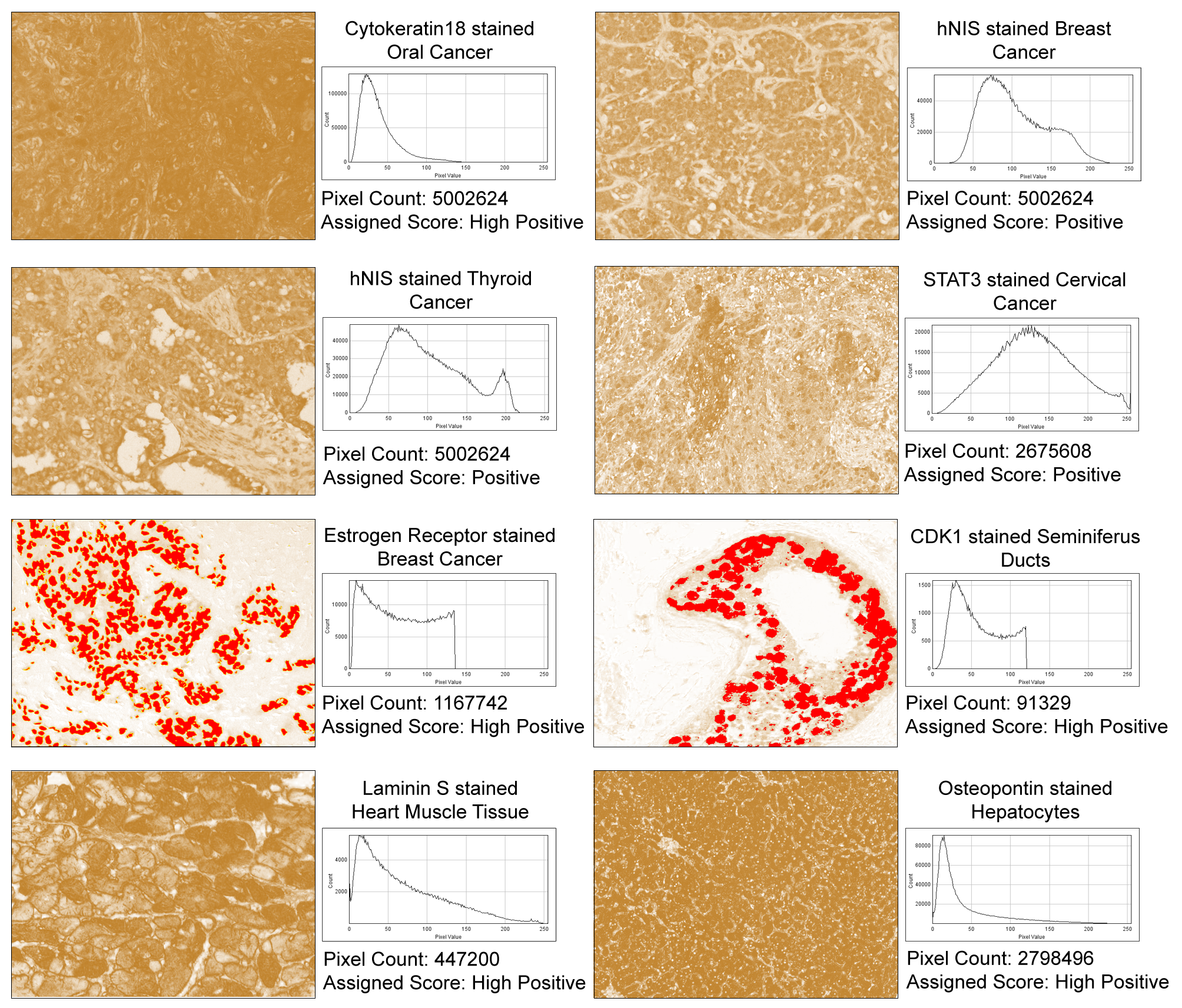

Supplement: Figure S2 — Demonstration of wide applicability of IHC profiler in various cancer and normal tissue types. Respective image analysis output and the score assigned using IHC Profiler is also shown for each image. Duly note, the varying number of pixels is solely due to the resolution of the microscope camera at which they were captured. (TIF) [file pone.0096801.s002.tif]

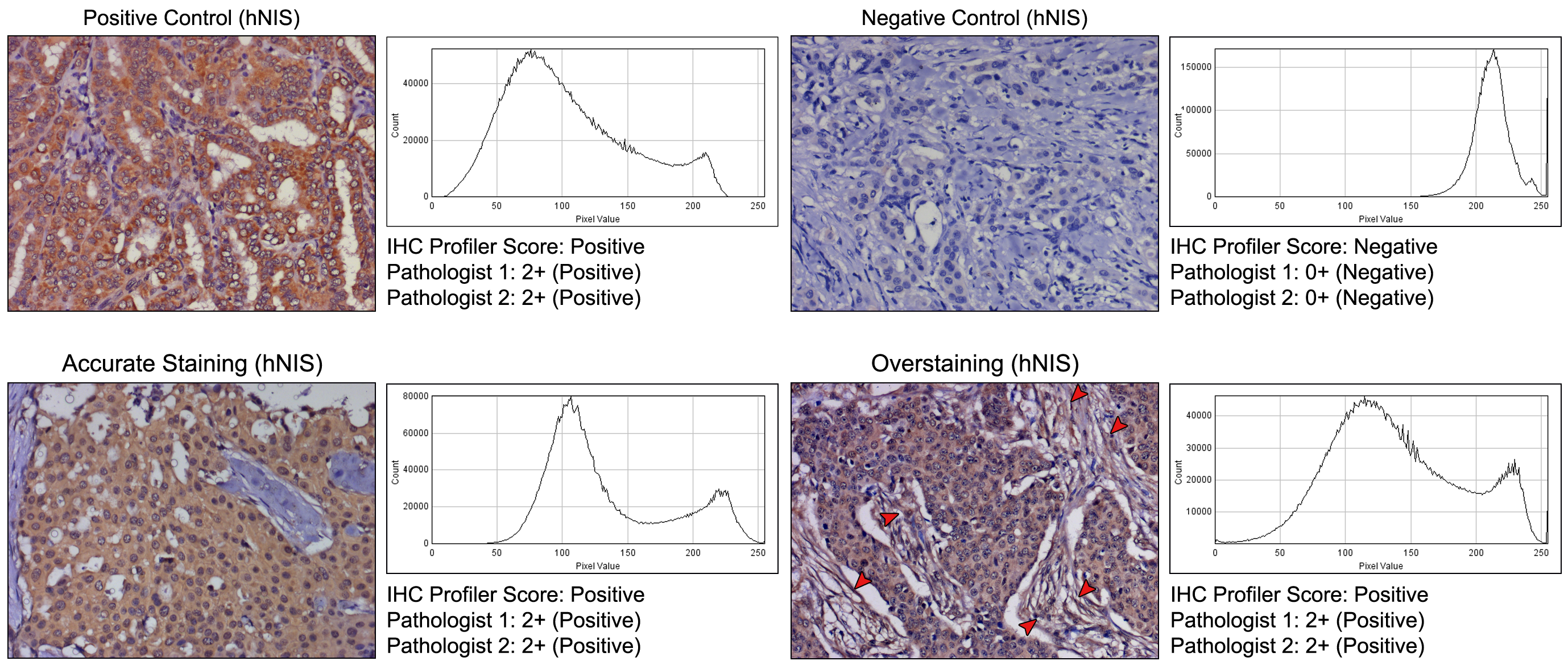

Supplement: Figure S3 — Qualitative measurement of IHC profiler accuracy. Qualitative measurement of IHC profiler accuracy and its comparison with pathological analysis post assessment of IHC profiler. Red arrow markings indicate overdeveloped regions of the sample as indicated by pathological assessment. (TIF) [file pone.0096801.s003.tif]
